# Maternal circulating Vitamin D_3_ levels during pregnancy and behaviour across childhood

**DOI:** 10.1038/s41598-019-51325-3

**Published:** 2019-10-15

**Authors:** Mónica López-Vicente, Jordi Sunyer, Nerea Lertxundi, Llúcia González, Cristina Rodríguez-Dehli, Mercedes Espada Sáenz-Torre, Martine Vrijheid, Adonina Tardón, Sabrina Llop, Maties Torrent, Jesús Ibarluzea, Mònica Guxens

**Affiliations:** 1 ISGlobal, Barcelona, Spain; 2 CIBER de Epidemiología y Salud Pública (CIBERESP), Madrid, Spain; 3 Universitat Pompeu Fabra (UPF), Barcelona, Spain; 4 Hospital del Mar Medical Research Institute (IMIM), Barcelona, Spain; 5 BIODONOSTIA, Donostia-San Sebastian, Spain; 6 University of the Basque Country (UPV/EHU), Donostia-San Sebastian, Spain; 7 Unidad Predepartamental de Medicina, UJI, Valencia, Spain; 8 Unidad Mixta de Investigación en Epidemiología y Salud Ambiental FISABIO-UV-UJI, Valencia, Spain; 9 Servicio de Pediatría, Hospital San Agustín, Avilés, Spain; 10 Public Health Laboratory, Basque Government, Bizkaia, Spain; 11 IUOPA-Preventive Medicine and Public Health Area, University of Oviedo and ISPA, Oviedo, Spain; 12 Menorca Health Area, Menorca, Spain; 13 Departamento de Salud del Gobierno Vasco, Gipuzkoa, Spain; 14 Department of Child and Adolescent Psychiatry/Psychology, Erasmus University Medical Centre-Sophia Children’s Hospital, Rotterdam, The Netherlands

**Keywords:** Paediatric research, Epidemiology, Nutrition, Human behaviour

## Abstract

Vitamin D deficiency during critical periods of development could lead to persistent brain alterations. We aimed to assess the association between maternal vitamin D_3_, the major circulatory form of vitamin D, at pregnancy and neurodevelopmental outcomes during childhood, namely: behavioural problems, Attention Deficit and Hyperactivity Disorder (ADHD) and Autism Spectrum Disorder (ASD) symptoms, and social competence. This study included 2,107 mother-child pairs of a Spanish population-based birth cohort. Maternal plasma vitamin D_3_ was measured in pregnancy. The outcomes were measured through questionnaires at 5, 8, 14, and 18 years old. We ran multivariate regression models adjusted for potential confounding variables. We found that per each 10 ng/mL increment of maternal vitamin D_3_, children obtained higher social competence scores (coefficient = 0.77; 95% CI = 0.19, 1.35) at 5 years old. However, we observed null associations between maternal vitamin D_3_ and total behavioural problems and ADHD and ASD symptoms in children from 5 to 18 years old. Further studies carried out in countries where the population is exposed to lower vitamin D levels are needed.

## Introduction

Vitamin D is a hormone that is involved in the formation of bone and in the regulation of several functions throughout the body, including the central nervous system function^1^. There are two forms of vitamin D, depending on the source: D_2_, of plant origin, and D_3_, of animal origin^1^. The majority of circulating vitamin D is in D_3_ form^2,3^, which is mainly produced photochemically in the epidermis by action of ultraviolet light^1,4^ and a small proportion comes from diet, principally from fish, eggs, and milk^5^. Vitamin D from the skin and diet is metabolized in the liver to 25-hydroxy-vitamin D (25[OH]D), the circulating form, which is one of the most stable vitamin D biomarkers, and in the kidney to 1,25-(OH)D, the active form^1^. During gestation, the foetus is entirely dependent on the maternal supply of 25(OH)D^6^.

Vitamin D modulates the central nervous system function mainly through its nuclear hormone receptor, the vitamin D receptor, which is expressed in neuronal and glial cells in almost all regions of the central nervous system^7^. It controls neuronal differentiation and maturation, regulates the genetic expression of neurotransmitters, and it has neuroprotective properties and antioxidant effects^1^. Vitamin D deficiency is a public health problem worldwide, particularly for darker skinned individuals living further away from the equator^8^. If this deficiency occurs during critical periods of development, it could lead to changes that persist through adulthood, and increases the risk of different psychiatric and neurological disorders, such as schizophrenia, multiple sclerosis, dementia, cognitive decline, Parkinson disease, and depression^9^. Some studies have investigated the association between vitamin D concentrations during pregnancy and neurodevelopmental outcomes, such as behavioural problems and social competence, in the offspring. Three independent studies observed no associations between maternal 25(OH)D measured in blood at 18, 26, and 33 weeks of pregnancy, respectively, and behavioural problems during childhood^9–11^. However, a recent study observed that higher maternal 25(OH)D concentration (>20.4 ng/mL) at week 13 of pregnancy was related to a lower number of behavioural problems and attention deficit and hyperactivity disorder (ADHD) symptoms at 4 years old^12^. Two other studies, one of them developed in our cohort, the Spanish population-based birth cohort INMA (INfancia y Medio Ambiente [Childhood and Environment]), also reported negative associations between prenatal 25(OH)D_3_ and ADHD symptoms at preschool age^13,14^. The evidence for an association between prenatal levels of 25(OH)D and autism spectrum disorder (ASD) symptoms is inconsistent, while some studies found negative relationships^6,15–19^, others obtained null results^20,21^. Nevertheless, a recently conducted meta-analysis showed negative associations between prenatal 25(OH)D_3_ and both ADHD and ASD symptoms^22^. Moreover, low concentrations of 25(OH)D during pregnancy have shown to be associated with delayed social development in toddlers^23,24^. Unlike the other mentioned outcomes, this domain is not considered a clinical phenotype, however, it encompasses several aspects of adaptive behavioural development with long-term consequences, such as unemployment, mental health problems, marital difficulties, delinquency, or violence^25^.

Further research on the association between prenatal 25(OH)D concentration and behaviour during childhood is needed. This study aims to assess the association between maternal circulating vitamin D_3_ (25[OH]D_3_) in pregnancy, when key neuronal maturation processes take place in the foetus, and behavioural problems, ADHD and ASD symptoms, and social competence from 5 to 18 years old in the INMA cohort, which is based in different regions of Spain.

## Results

The characteristics of the entire sample and by the three clinically relevant categories of maternal 25(OH)D_3_ concentration in plasma during pregnancy are described in Supplementary Table S1. The study population included 50.8% male children and 56.4% firstborn. Ninety-four percent of the mothers were born in Spain, and maternal mean age was 30.7 (4.2) years old. Twenty-five percent of the mothers were overweight or obese before pregnancy, 25.8% had a low education level (primary or less) and 34% had high education level (university). Half of the mothers had a manual occupation, 17% of the mothers were smokers during the first trimester of pregnancy, and 29% reported that their partners smoked at home. Eighteen percent of the women (n = 372) had deficient concentrations of 25(OH)D_3_ (<20 ng/mL) during pregnancy. The highest concentrations of circulating 25(OH)D_3_ were observed in Valencia (mean [SD] 32.8 [10.8] ng/mL), and the lowest, in Asturias (mean [SD] 28 [10.2] ng/mL). Higher concentrations of 25(OH)D_3_ were detected in women from high education level and from managers/technicians occupation category, and those born in Europe, including Spain, as opposed to those women born in Latin America. Lower 25(OH)D_3_ concentrations were measured in younger women and in women with pre-pregnancy overweight or obesity, primiparous, active smokers and with partners smoking at home during pregnancy.

Behavioural problems, ADHD symptoms, ASD symptoms, and social competence were assessed using different instruments and at different children’s ages in each of the regions (Supplementary Table S2). Supplementary Table S3 shows the distribution of the outcomes included in this study according to the categories of maternal 25(OH)D_3_. Children prenatally exposed to deficient concentrations of 25(OH)D_3_ (<20 ng/mL) showed more ASD symptoms, more total behavioural problems, and less social competence at 5 years old, as compared to their peers exposed to higher concentrations. This group of children with low concentrations of 25(OH)D_3_ during gestation had more ADHD symptoms at 8 years old and more total behavioural problems at 8 and at 14 years old. However, this pattern was not observed at 18 years old.

Table 1 shows the associations between prenatal 25(OH)D_3_ concentrations and the neurodevelopmental outcomes. Regarding the outcomes measured at 5 years old, the regression models indicated that sufficient 25(OH)D_3_ concentrations during pregnancy (≥30 ng/ml) were associated with lower ASD symptoms (coefficient = −0.54; 95% CI = −1.01, −0.07) compared to deficient 25(OH)D_3_ concentrations, and that insufficient concentrations (20–29.9 ng/ml) were associated with lower total behavioural problems measured with the Strengths and Difficulties Questionnaire (SDQ) (coefficient = −1.65; 95% CI = −3.27, −0.03) compared to deficient 25(OH)D_3_ concentrations, although these associations were no longer present in the fully adjusted models. The association with social competence remained after adjusting for potential confounding variables, using the exposure variable as continuous and categorical forms. Per each 10 ng/mL increment of maternal 25(OH)D_3_ concentration in pregnancy, children obtained 0.77 (95% CI = 0.19, 1.35) points more of social competence. Similarly, the score was 2.14 (95% CI = 0.47, 3.82) points higher in children with sufficient prenatal concentrations of 25(OH)D_3_ versus children with deficient concentrations. A positive association was also detected in the insufficient category (coefficient = 2.09; 95% CI = 0.33, 3.84), which was not observed in the fully adjusted model. The associations observed between maternal 25(OH)D_3_ in pregnancy, treated as continuous and categorical variable, and total behavioural problems and ADHD symptoms in the offspring at 8, 14, and 18 years old were almost null. We observed that insufficient 25(OH)D_3_ concentrations (20–29.9 ng/mL) category was associated with lower total behavioural problems score measured with the Child Behaviour Checklist (CBCL) (Incidence Rate Ratio [IRR] = 0.86; 95% CI = 0.76, 0.97), compared to de deficiency category (<20 ng/ml), but this association was no longer found after adjusting for potential confounding variables.

**Table 1:** Associations between maternal 25(OH)D_3_ concentrations in pregnancy and neurodevelopmental outcomes.

Domains	Instruments	Regions	Levels (n)	Unadjusted	Minimally adjusted^c^	Fully adjusted^d^
ASD symptoms	CAST-5y^a^ (coef, 95% CI)	V, S, A, G	Continuous (per 10 ng/mL) (1510)	−0.14 (−0.29, 0.01)	−0.10 (−0.25, 0.04)	−0.02 (−0.17, 0.12)
			<20 ng/mL (259)	Reference		
			20–29.9 ng/mL (534)	−0.23 (−0.72, 0.26)	−0.25 (−0.72, 0.22)	−0.14 (−0.61, 0.32)
			≥30 ng/mL (717)	**−0.54 (−1.01, −0.07)**	**−0.48 (−0.94, −0.02)**	−0.26 (−0.71, 0.19)
Social competence	CPSCS-5y^a^ (coef, 95% CI)	V, S, A, G, M	Continuous (per 10 ng/mL) (1481)	**1.22 (0.63, 1.81)**	**0.97 (0.39, 1.55)**	**0.77 (0.19, 1.35)**
			<20 ng/mL (279)	Reference		
			20–29.9 ng/mL (538)	**2.09 (0.33, 3.84)**	**1.97 (0.24, 3.69)**	1.42 (−0.29, 3.12)
			≥30 ng/mL (664)	**3.42 (1.72, 5.12)**	**2.84 (1.16, 4.52)**	**2.14 (0.47, 3.82)**
Total behavioural problems	SDQ-5y^a^ (coef, 95% CI)	G	Continuous (per 10 ng/mL) (263)	−0.24 (−0.78, 0.30)	—	−0.01 (−0.53, 0.51)
			<20 ng/mL (39)	Reference		
			20–29.9 ng/mL (124)	**−1.65 (−3.27, −0.03)**	—	−0.90 (−2.44, 0.63)
			≥30 ng/mL (100)	−1.02 (−2.70, 0.65)	—	−0.13 (−1.75, 1.48)
Total behavioural problems	SDQ-8y^a^ (coef, 95% CI)	V, S, A, G	Continuous (per 10 ng/mL) (1622)	−0.08 (−0.32, 0.15)	−0.13 (−0.37, 0.10)	0.00 (−0.23, 0.24)
			<20 ng/mL (288)	Reference		
			20–29.9 ng/mL (576)	−0.44 (−1.16, 0.29)	−0.51 (−1.24, 0.22)	−0.31 (−1.02, 0.41)
			≥30 ng/mL (758)	−0.44 (−1.14, 0.26)	−0.60 (−1.30, 0.11)	−0.22 (−0.91, 0.48)
Total behavioural problems	CBCL-8y^b^ (IRR, 95% CI)	V, S, G	Continuous (per 10 ng/mL) (1206)	0.98 (0.95, 1.02)	0.98 (0.94, 1.02)	1.00 (0.96, 1.04)
			<20 ng/mL (202)	Reference		
			20–29.9 ng/mL (415)	**0.86 (0.76, 0.97)**	**0.87 (0.77, 0.99)**	0.90 (0.79, 1.01)
			≥30 ng/mL (589)	0.92 (0.82, 1.04)	0.91 (0.81, 1.02)	0.96 (0.85, 1.07)
ADHD symptoms	CPRS-8y^b^ (IRR, 95% CI)	V, S, A, G	Continuous (per 10 ng/mL) (1622)	1.01 (0.97, 1.05)	1.00 (0.96, 1.04)	1.01 (0.97, 1.06)
			<20 ng/mL (288)	Reference		
			20–29.9 ng/mL (576)	0.93 (0.82, 1.06)	0.93 (0.81, 1.05)	0.94 (0.83, 1.07)
			≥30 ng/mL (758)	0.96 (0.85, 1.09)	0.94 (0.83, 1.06)	0.98 (0.86, 1.11)
Total behavioural problems	SDQ-14y^a^ (coef, 95% CI)	M	Continuous (per 10 ng/mL) (198)	−0.46 (−1.13, 0.21)	—	−0.27 (−0.95, 0.41)
			<20 ng/mL (34)	Reference		
			20–29.9 ng/mL (82)	−0.81 (−2.59, 0.97)	—	−0.43 (−2.23, 1.37)
			≥30 ng/mL (82)	−1.13 (−2.91, 0.66)	—	−0.64 (−2.45, 1.17)
Total behavioural problems	SDQ-18y^a^ (coef, 95% CI)	M	Continuous (per 10 ng/mL) (149)	−0.03 (−0.80, 0.73)	—	0.09 (−0.69, 0.86)
			<20 ng/mL (24)	Reference		
			20–29.9 ng/mL (58)	1.11 (−0.96, 3.18)	—	1.48 (−0.61, 3.58)
			≥30 ng/mL (67)	0.54 (−1.49, 2.57)	—	0.92 (−1.16, 2.99)
ADHD symptoms	CPRS-18y^b^ (IRR, 95% CI)	M	Continuous (per 10 ng/mL) (151)	0.96 (0.81, 1.14)	—	0.96 (0.80, 1.14)
			<20 ng/mL (25)	Reference		
			20–29.9 ng/mL (60)	1.45 (0.93, 2.27)	—	1.33 (0.84, 2.10)
			≥30 ng/mL (66)	1.22 (0.79, 1.90)	—	1.13 (0.72, 1.77)

*Domains*: ADHD = Attention Deficit and Hyperactivity Disorder; ASD = Autism Spectrum Disorder. *Instruments*: CAST = Childhood Autism Spectrum Test; CBCL = Child Behaviour Checklist; CPRS = Conners’ Parent Rating Scale-Revised (short form); CPSCS = California Preschool Social Competence Scale; SDQ = Strengths and Difficulties Questionnaire. *Regions*: A = Asturias; G = Gipuzkoa; M = Menorca; S = Sabadell; V = Valencia. ^a^Linear regression model; ^b^Negative binomial regression model; ^c^Adjusted for region; ^d^Adjusted for region, maternal age, maternal education level, maternal occupation, maternal country of birth, maternal smoking during the 1^st^ trimester of pregnancy, partner smoking at home during the 1^st^ trimester of pregnancy. Multiple imputation and inverse probability weighting were applied. Bold = p < 0.05.

As sensitivity analysis, no associations were detected between child 25(OH)D_3_ concentrations and the outcomes in the subsample of children with available child 25(OH)D_3_ concentrations. The association between prenatal 25(OH)D_3_ and child social competence remained when we restricted the analysis to the sample of children with both prenatal and child 25(OH)D_3_ data available (Supplementary Table S4).

## Discussion

We explored the associations between maternal 25(OH)D_3_ concentration during pregnancy and a wide range of behavioural problems, including ADHD and ASD symptoms, and social competence in children at different ages using data from a population-based birth cohort located in five regions across Spain. Our findings indicated a positive association between prenatal 25(OH)D_3_ concentrations and social competence at 5 years of age. However, we observed null associations of prenatal 25(OH)D_3_ concentration with total behavioural problems, ADHD and ASD symptoms in children from 5 to 18 years old. Sensitivity analyses showed no associations between the concentrations of 25(OH)D_3_ in children and the outcomes.

In our study, we observed a weak negative association between prenatal 25(OH)D_3_ and ASD symptoms at 5 years of age that was not significant upon adjustment for potential confounding variables. These results are in line with two Californian studies^20,21^. On the contrary, previous research carried out in Australian^15^ and Dutch birth cohort studies^6,19^ found stronger associations with this outcome. The Australian study reported an association between lower mid-gestation 25(OH)D concentrations and higher scores on the Attention Switching subscale of the Autism-Spectrum Quotient^15^. In the Dutch studies, infants exposed to persistent low 25(OH)D concentrations (<7.9 ng/mL) from mid-gestation until birth showed more autism-related traits at 6 years old^19^, and mid-gestational 25(OH)D deficiency (<7.9 ng/mL) was associated with an increased risk of ASD diagnosis at 9 years old^6^. The different findings obtained could be explained by the lower levels of 25(OH)D in Dutch pregnant women, as compared to the Spanish sample. Other plausible explanations are the different pregnancy periods at blood collection and the different instruments used to assess ASD symptoms. The instrument used in the Dutch studies, the Social Responsiveness Scale, is particularly focused on measuring the social deficits associated with ASD^26^. This suggests that prenatal vitamin D could be more related to the social dimension of ASD. The previously reported positive association between prenatal vitamin D and social development in toddlers^23,24^ together with our findings on social competence at age 5 years reinforces this hypothesis. In our study, we obtained a relatively low effect estimates for the association between prenatal vitamin D levels and social competence. However, this effect size is in line with other studies assessing other exposures, such as head circumference^27^, child salivary cortisol levels at 14 months of age^28^, and maternal urinary phthalate metabolite levels during pregnancy^29^. Furthermore, although it might not be relevant at a clinical level, the observed effect may have an impact at a population level. If the whole population is exposed to low levels of vitamin D, the distribution of social competence scores would likely move to the left, and the prevalence of children with low social competence would increase substantially. This increase of social competence problems may have a negative impact on the community in terms of unemployment, mental health problems or delinquency^25^.

We observed no associations between the concentrations of 25(OH)D_3_ in children and the assessed outcomes in a subsample, while the association remained between prenatal 25(OH)D_3_ and social competence in the same group. This finding suggests that the levels of 25(OH)D_3_ influence child social development exclusively during the prenatal period. The biological mechanisms by which maternal vitamin D concentration during pregnancy affect the brain development in humans are still unclear. However, some animal studies observed that 25(OH)D_3_ deficiency during late gestation was associated with brain morphological alterations in the offspring, such as larger lateral ventricles, thinner cortex, and higher cell proliferation^30^, as well as hyperlocomotion at adulthood^31^. Although the most active period of foetal brain growth lies in the third trimester of pregnancy, key development processes, such as neuronal migration, start at week 6 of gestation and usually end around 24 weeks, and vitamin D is involved in the modulation of neuronal migration, dendritic spine morphology, and neuronal connectivity^1^. Therefore, the association we observed between 25(OH)D_3_ concentration in pregnancy and child social competence could be due to alterations in these neuronal processes.

The null associations detected between maternal 25(OH)D_3_ concentration in pregnancy and total behavioural problems in the offspring are consistent with some previous studies^9–11^. However, Daraki *et al*.^12^ found that higher 25(OH)D levels (>20.4 ng/mL) were related to decreased number of total behavioural problems in 4-year-old children^12^. In this study, carried out in a Greek cohort, almost two-thirds of pregnant women (n = 313) had 25(OH)D deficiency (<15.7 ng/mL), which seemed to be explained by darker skin pigmentation, poor dietary vitamin D intake, veiled clothing reducing sunshine exposure, and increased prevalence of obesity. This high prevalence of 25(OH)D deficiency could explain the different results obtained between this study and ours.

Regarding ADHD symptoms, we found that, despite the associations already observed between prenatal 25(OH)D_3_ and ADHD at preschool age in our cohort^13^ and in the Danish^14^ and the Greek^12^ cohorts, these associations did not remain at 8 and 18 years old. A possible explanation is that prenatal 25(OH)D_3_ concentrations do not have permanent effects on the brain development, but other factors could modulate these effects across the childhood and adolescence, exerting much more influence on ADHD symptoms at 8 and 18 years old than prenatal 25(OH)D_3_. Alternative explanations to the inconsistent findings between preschool age and older age in our cohort include the different instruments used in both periods and the different person reporting ADHD symptoms. At ages 4–5 years, we administered the ADHD Criteria of Diagnostic and Statistical Manual of Mental Disorders (ADHD-DSM-IV) form to the teachers, while at 8 and 18 years old the parents filled in the Conners’ Parent Rating Scale-Revised (short form) (CPRS-R [S]). It is known that agreement between parents and teachers regarding child ADHD symptoms is relatively poor^32^. However, previous research performed in a Scandinavian study using clinical ADHD diagnosis also reported null associations between prenatal 25(OH)D concentrations and ADHD at older ages^33,34^, which supports the hypothesis about the reversibility of the prenatal vitamin D deficiency effects on brain development.

This study has some limitations. First, we only used D_3_ form of vitamin D, however this is the major circulatory form and is commonly used in endocrinology studies^3,30,31^. Second, a single 25(OH)D_3_ measurement per subject was obtained, so it was not possible to compare different windows of susceptibility to 25(OH)D_3_ deficiency during pregnancy. Third, the heterogeneity in the assessment periods between regions complicated the interpretation of the results. Due to the small sample size of adolescents in our study, results on long-term associations between prenatal 25(OH)D_3_ concentrations and behavioural problems at 14 and 18 years old should be taken with caution. Moreover, given the prospective nature of the study, we had some loss of participants across follow ups. This limitation was minimized by applying inverse probability weighting in all the analyses. We also performed several tests that could have increased the risk of chance findings or Type I errors, however the association we observed was robust, given the consistency in the direction of the associations between the models, unadjusted and adjusted, and using the exposure as continuous and as categorical variable, and in line with previous literature. In addition, lack of information on parental behavioural problems during pregnancy could have resulted in some residual confounding.

The main strengths of this study include its population-based, prospective design, and large sample size located in heterogeneous geographical areas. Therefore, the conclusions of the study are generalizable to the general population. Several methodological aspects are also considered major strengths of the study, such as the inclusion of important confounding variables in the statistical models, the multiple imputation of missing covariate values, the use of inverse probability weighting to control the potential selection bias induced by loss of follow-up. Finally, the inclusion of several behavioural problems domains and ages conferred a wide view of the role of prenatal 25(OH)D_3_ on neurodevelopmental alterations, not restricted to specific types of behavioural problems or focused on single age periods.

In conclusion, we observed that prenatal 25(OH)D_3_ was related to social skills at 5 years of age. On the contrary, we found no evidence for the association between prenatal 25(OH)D_3_ and several behavioural problems across childhood including ADHD and ASD symptoms. Further studies carried out in countries where the population is exposed to lower vitamin D levels are needed.

## Methods

### Study population

**Figure 1:**
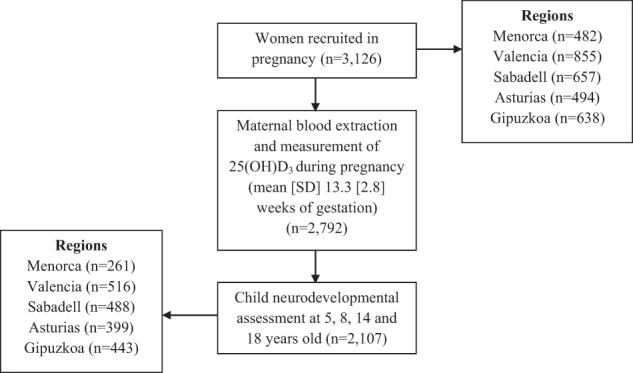
Flowchart of study population.

This study was embedded in the INMA Project, a population-based birth cohort based in different regions across Spain^35^. For the current study, we used data from Menorca (40°N), Valencia (39°N), Sabadell (41°N), Asturias (43°N), and Gipuzkoa (43°N) regions. A total of 3,126 pregnant women were recruited at their first routine specialized antenatal care visit (10–13 weeks of gestation) in the main public hospital or reference health centre. The recruitment took place between February 1997 and September 1998 in Menorca, and between November 2003 and February 2008 in the other regions. Since then, we collected data from the participants every two years approximately. The 89.3% (2,792) of the baseline sample had 25(OH)D_3_ data available. A total of 2,107 mother-child pairs, who had 25(OH)D_3_ measured and at least one of the behavioural assessments available, were included in this study, representing the 67.4% of the initial sample (Fig. 1).

This study was approved by the Clinical Research Ethical Committees of the Asturias, Donostia (Gipuzkoa), and La Fe (Valencia) Hospitals, and the Medical Assistance Municipal Institute (Barcelona), all research was performed in accordance with the relevant guidelines and regulations, and written informed consent was obtained from all participants for each phase.

### 25(OH)D_3_ assessment

A single maternal blood specimen was drawn during pregnancy (mean [SD] 13.3 [2.8] weeks of gestation). Most blood draws (78.6%) were done during the second trimester of pregnancy, with 20.6% during the first trimester, and 0.8% during the third trimester. We also measured child plasma concentrations of 25(OH)D_3_ in a subgroup of 808 participants (mean [SD] 4.5 [0.2] years old). Samples were processed immediately and stored from −70 to −80 °C until analysis. Plasma concentrations of 25(OH)D_3_ were quantified by high-performance liquid chromatography method using a BioRAD kit according to Clinical and Laboratory Standard Institute protocols^36^. Detection limit was 5 ng/ml, and interassay coefficient of variation was 4.5%. The assay was validated by German Programes of External Evaluation of Quality (DGKL-RFB-Referenzinstitut für Bionalytik), and results were satisfactory in 100% of the cases.

### Neurodevelopmental outcomes assessment

ASD symptoms were measured through the Childhood Autism Spectrum Test (CAST)^37^. A psychologist administered this 37-item questionnaire to the child’s parents. The CAST identifies subtle manifestations of autism spectrum conditions (social impairments, communication impairments, and repetitive or stereotyped behaviours). The total score ranges from 0 to 31 points.

Social competence was reported by teachers using the California Preschool Social Competence Scale (CPSCS)^38^. The CPSCS consists of 30 items that result into scores for five subscales (considerateness, task orientation, extraversion, verbal facility, and response to unfamiliar) and a general social competence score. The scores were standardized to a mean of 100 and a standard deviation of 15.

Behavioural problems were reported by parents using SDQ^39^ and CBCL^40^. The SDQ consists of 25 items with five for each dimension: emotional problems, conduct problems, hyperactivity/inattention problems, peer/social problems, and pro-social behaviour. The sum of the four problematic scores (excluding pro-social behaviour) yields a total behavioural problems score, ranging from 0 to 40. Regarding the CBCL, we used the version for children between 6 and 18 years old, comprised by 113 items that measure nine dimensions: anxious/depressed, withdrawn/depressed, somatic complaints, social problems, thought problems, attention problems, rule-breaking behaviour, aggressive behaviour, and other problems. The sum of all subscales’ scores yields a total behavioural problems score, ranging from 0 to 240.

ADHD symptoms were reported by parents using the CPRS-R (S)^41^. This questionnaire comprises 27 items that result into scores for three subscales (oppositional, cognitive problems/inattention, hyperactivity), and a general ADHD index, ranging from 0 to 36.

### Covariates assessment

During the first trimester of pregnancy, we used questionnaires to collect maternal background information, such as age, country of birth (Spain, Latin America, Europe, and other country), education level (primary or lower, secondary, and university degree), social class (managers/technicians, non-manual, and manual occupation)^42^, parity (0, 1, 2 or more), and pre-pregnancy body mass index (BMI) based on measured height at recruitment and pre-pregnancy self-reported weight (kilograms per square meter, kg/m^2^; underweight [<18.5], normal weight [18.5, <25], overweight [25, <30], and obese [≥30]). We collected information on active smoking and partner smoking at home during the first trimester of pregnancy.

### Statistical analysis

The concentrations of 25(OH)D_3_ are highly dependent on the season when they are measured. To take into consideration the seasonal distribution of 25(OH)D_3_ and to reduce the influence of competing exposures not related to 25(OH)D_3_ status (e.g. winter infection, melatonin-related exposures, etc.), we deseasonalized the variable. We first tested seasonality of 25(OH)D_3_ concentration by fitting the data to a sine function with a period of 12 months in a nonlinear regression cosinor model^43^. We subtracted the predicted concentrations based on month at blood collection from the actual observed value for each subject. In our analyses, we used the residuals derived from the sinusoidal model, centred to the overall mean, as the exposure variable. Maternal and child 25(OH)D_3_ concentrations were treated as continuous (effect per 10 ng/ml increment) and as categorical divided into clinically relevant categories (<20 ng/ml [deficiency, reference group], 20 to 29.9 ng/ml [insufficiency], and ≥30 ng/ml [sufficiency]). Although there is no absolute consensus regarding the cut-offs that should be used, most experts agree on these categories^44^.

We ran linear regression models to examine the association between maternal 25(OH)D_3_ concentration in pregnancy and ASD symptoms, social competence, and total behavioural problems measured with the SDQ, as continuous scores. Negative binomial regression models were used for total behavioural problems measured with the CBCL and ADHD symptoms as continuous scores. Regression models were performed unadjusted, minimally adjusted (for region), and fully adjusted (for maternal age, maternal education level, maternal occupation, maternal country of birth, maternal smoking during pregnancy, and partner smoking at home during pregnancy). The confounding variables were defined based on scientific literature and on availability of data within the INMA Project.

We performed multiple imputations of missing values using chained equations to impute missing potential confounding variables among all participants with available data on the exposure and the outcomes. We generated and separately analysed twenty-five completed data sets and we combined the results using the standard Rubin’s rules^45^, assuming missing at random data.

Children included in the analysis (n = 2,107) were more likely to have parents from a higher socioeconomic position compared to children that were not included due to missing data in either the exposure or one of the outcomes (n = 1,019). We therefore applied inverse probability weighting (IPW) to correct for selection bias that potentially arises when only population with available exposure and outcome data is included as compared to the full initial cohort recruited at pregnancy^46^.

As sensitivity analyses, we repeated all the models using child 25(OH)D_3_ concentration as exposure variable, instead of maternal concentrations during pregnancy, in order to assess the associations between postnatal 25(OH)D_3_ and the outcomes.

We performed all the statistical analyses using Stata 12.0 (Stata Corporation, College Station, Texas).

## Supplementary information

Supplementary tables

## Data Availability

The datasets generated during and/or analysed during the current study are not publicly available but are available from the corresponding author on reasonable request.
