# Supplementary material for: Maternal circulating Vitamin D3 levels during pregnancy and behaviour across childhood
Source: Sci Rep. 2019 Oct 15;9:14792. doi: 10.1038/s41598-019-51325-3 (PMC6794315; doi:10.1038/s41598-019-51325-3)
Supplement: Supplementary file 1 — Supplementary tables [file 41598_2019_51325_MOESM1_ESM.pdf]

Maternal circulating Vitamin D_3 levels during pregnancy and behaviour across childhood

Mónica López-Vicente, Jordi Sunyer, Nerea Lertxundi, Llúcia González, Cristina Rodríguez-Dehli, Mercedes Espada Sáenz-Torre, Martine Vrijheid, Adonina Tardón, Sabrina Llop, Maties Torrent, Jesús Ibarluzea, Mònica Guxens

Supplementary Tables (S1 to S4)

Supplementary Table S1. Characteristics of participants according to maternal 25(OH)D_3 concentrations in pregnancy

	25(OH)D_3-deficient (<20 ng/mL) (n=372)	25(OH)D_3-insufficient (20-29 ng/mL) (n=767)	25(OH)D_3-sufficient (>=30 ng/mL) (n=968)	Total (n=2107)	Missings (%)
**Region (n, %)**					0
Menorca	44 (11.8)	109 (14.2)	108 (11.2)	261 (12.4)	
Valencia	55 (14.8)	156 (20.3)	305 (31.5)	516 (24.5)	
Sabadell	109 (29.3)	155 (20.2)	224 (23.1)	488 (23.2)	
Asturias	93 (25.0)	153 (20.0)	153 (15.8)	399 (18.9)	
Gipuzkoa	71 (19.1)	194 (25.3)	178 (18.4)	443 (21.0)	
**Maternal age (mean, SD)**	30.41 (4.5)	30.54 (4.2)	31.02 (4.1)	30.74 (4.2)	0
**Child's sex (n, % male)**	196 (52.7)	389 (50.7)	486 (50.2)	1071 (50.8)	0
**Maternal education (n, %)**					0.5
Primary	111 (29.9)	194 (25.4)	235 (24.4)	540 (25.8)	
Secondary	154 (41.5)	311 (40.8)	378 (39.3)	843 (40.2)	
University	106 (28.6)	258 (33.8)	350 (36.3)	714 (34.1)	
**Maternal social class (n, %)**					3.2
Managers/technicians	67 (18.5)	162 (22.0)	234 (24.8)	463 (22.7)	
Non-manual	98 (27.1)	229 (31.2)	318 (33.7)	645 (31.6)	
Manual	197 (54.4)	344 (46.8)	391 (41.5)	932 (45.7)	
**Paternal social class (n, %)**					2.2
Managers/technicians	68 (18.7)	142 (19.1)	227 (23.8)	437 (21.2)	
Non-manual	78 (21.5)	201 (27.0)	227 (23.8)	506 (24.6)	
Manual	217 (59.8)	402 (54.0)	499 (52.4)	1118 (54.3)	
**Maternal country of birth (n, %)**					0.2
Spain	340 (91.6)	733 (95.8)	914 (94.6)	1987 (94.5)	
Latin America	22 (5.9)	24 (3.1)	30 (3.1)	76 (3.6)	
Europe	6 (1.6)	8 (1.1)	20 (2.1)	34 (1.6)	
Other	3 (0.8)	0 (0.0)	2 (0.2)	5 (0.2)	
**Paternal country of birth (n, %)**					0.2
Spain	347 (93.3)	722 (95.0)	897 (92.9)	1966 (93.5)	
Latin America	12 (3.2)	25 (3.3)	32 (3.3)	69 (3.3)	
Europe	6 (1.6)	13 (1.7)	27 (2.8)	46 (2.2)	
Other	7 (1.9)	4 (0.5)	10 (1.0)	21 (1.0)	

	**$25(OH)D_3$-deficient (<20 ng/mL) (n=372)**	**$25(OH)D_3$-insufficient (20-29 ng/mL) (n=767)**	**$25(OH)D_3$-sufficient (>=30 ng/mL) (n=968)**	**Total (n=2107)**	**Missings (%)**
**Maternal pre-pregnancy BMI (n, %)**					0.3
Underweight (<18.5)	24 (6.5)	30 (3.9)	33 (3.4)	87 (4.1)	
Normal weight (18.5, <25)	237 (64.1)	539 (70.3)	698 (72.4)	1474 (70.2)	
Overweight (25, <30)	79 (21.4)	144 (18.8)	158 (16.4)	381 (18.1)	
Obese (≥30)	30 (8.1)	54 (7.0)	75 (7.8)	159 (7.6)	
**Maternal parity (n, %)**					0.2
0	218 (59.1)	431 (56.3)	537 (55.5)	1186 (56.4)	
1	129 (35.0)	294 (38.4)	359 (37.1)	782 (37.2)	
≥2	22 (6.0)	41 (5.4)	71 (7.3)	134 (6.4)	
**Maternal smoking during 1st trimester of pregnancy (n, %)**	81 (22.3)	125 (16.7)	146 (15.2)	352 (17.0)	1.8
**Partner smoking at home during 1st trimester of pregnancy (n, %)**	123 (33.8)	215 (29.0)	266 (27.8)	604 (29.3)	2.1

BMI=body mass index; SD=standard deviation.

Supplementary Table S2. Instruments used to measure the outcome domains and children's ages when they were applied for each region

Domain	Instrument	Evaluator	Age			
			5 years	8 years	14 years	18 years
ASD symptoms	CAST	Psychologist	A, G, S, V			
Social competence	CPSCS	Teachers	A, G, M, S, V			
Behavioural problems	SDQ	Parents	G	A, G, S, V	M	M
	CBCL	Parents		G, S, V		
ADHD symptoms	CPRS	Parents		A, G, S, V		M

Domains: ADHD=Attention Deficit and Hyperactivity Disorder; ASD=Autism Spectrum Disorder. *Instruments*: CAST=Childhood Autism Spectrum Test; CBCL=Child Behaviour Checklist; CPRS=Conners' Parent Rating Scale-Revised (short form); CPSCS=California Preschool Social Competence Scale; SDQ=Strengths and Difficulties Questionnaire.
Regions: A=Asturias; G=Gipuzkoa; M=Menorca; S=Sabadell; V=Valencia.

Supplementary Table S3. Distribution of outcome scores according to maternal 25(OH)D_3 concentrations in pregnancy

Domains	Instruments	**25(OH)D_3-deficient (<20 ng/mL)**	**25(OH)D_3-insufficient (20-29 ng/mL)**	**25(OH)D_3-sufficient (>=30 ng/mL)**
ASD symptoms	CAST-5y (n=1510) (mean, SD)	6.53 (3.31)	6.31 (3.35)	5.97 (3.14)
Social competence	CPSCS-5y (n=1481) (mean, SD)	93.29 (12.61)	95.27 (13.06)	96.65 (11.16)
Behavioural problems	SDQ-5y (n=263) (mean, SD)	9.79 (4.96)	8.19 (4.41)	8.82 (4.54)
Behavioural problems	SDQ-8y (n=1622) (mean, SD)	9.10 (5.11)	8.71 (5.13)	8.76 (5.06)
Behavioural problems	CBCL-8y (n=1206) (mean, SD)	28.72 (20.30)	24.90 (18.35)	26.67 (19.30)
ADHD symptoms	CPRS-8y (n=1622) (mean, SD)	8.39 (7.43)	7.76 (6.79)	8.03 (7.06)
Behavioural problems	SDQ-14y (n=198) (mean, SD)	10.38 (4.22)	9.57 (4.36)	9.26 (4.59)
Behavioural problems	SDQ-18y (n=149) (mean, SD)	9.25 (3.91)	9.97 (4.04)	9.27 (4.42)
ADHD symptoms	CPRS-18y (n=151) (mean, SD)	4.96 (4.41)	7.00 (6.63)	5.82 (5.83)

Domains: ADHD=Attention Deficit and Hyperactivity Disorder; ASD=Autism Spectrum Disorder. *Instruments*: CAST=Childhood Autism Spectrum Test; CBCL=Child Behaviour Checklist; CPRS=Conners' Parent Rating Scale-Revised (short form); CPSCS=California Preschool Social Competence Scale; SDQ=Strengths and Difficulties Questionnaire.

Supplementary Table S4. Associations between child 25(OH)D_3 concentrations at 4 years old and maternal 25(OH)D_3 concentrations in pregnancy and social competence[a] in a subsample (n=597)

**Measurement period**	**Regions**	**Levels (n)**	**Fully adjusted (coef, 95%CI)[b]**
Child 25(OH)D_3	V, S, A, G	Continuous (per 10 ng/mL) (597)	-0.22 (-1.07, 0.64)
		<20 ng/mL (153)	Reference
		20-29.9 ng/mL (209)	-0.29 (-2.68, 2.11)
		≥30 ng/mL (235)	-1.22 (-4.05, 1.62)
Maternal 25(OH)D_3	V, S, A, G	Continuous (per 10 ng/mL) (597)	0.76 (-0.07, 1.60)
		<20 ng/mL (126)	Reference
		20-29.9 ng/mL (222)	**4.20 (1.80, 6.59)**
		≥30 ng/mL (249)	**3.39 (1.03, 5.74)**

Regions: A=Asturias; G=Gipuzkoa; S=Sabadell; V=Valencia. [a]California Preschool Social Competence Scale (CPSCS); [b]Linear regression model adjusted for region, maternal age, maternal education level, maternal occupation, maternal country of birth, maternal smoking during pregnancy, partner smoking at home during pregnancy. Multiple imputation and inverse probability weighting were applied. Bold = $p<0.05$.
